# Postnatal quality of life in women after normal vaginal delivery and caesarean section

**DOI:** 10.1186/1471-2393-9-4

**Published:** 2009-01-30

**Authors:** Behnaz Torkan, Sousan Parsay, Minoor Lamyian, Anoshirvan Kazemnejad, Ali Montazeri

**Affiliations:** 1 Faculty of Nursing and Midwifery, Khorasgan Azad University, Isfahan, Iran; 2 Department of Health and Social Medicine, Shahid Beheshti University of Medical Sciences, Tehran, Iran; 3 Faculty of Medicine, Tarbiat Modares University, Tehran, Iran; 4 Iranian Institute for Health Sciences Research, ACECR, Tehran, Iran

## Abstract

**Background:**

Caesarean section might increase the incidence of surgical interventions and problems resulting from hospitalization and thus affecting quality of life in women after delivery. This study aimed to compare quality of life in women after normal delivery and caesarean section.

**Methods:**

This was a prospective study. A sample of women with normal delivery and caesarean section from 5 health care centers in Isfahan, Iran were entered into the study. Quality of life was measured using the SF-36 at two points in time (time 1: 6 to 8 weeks after delivery; time 2: 12 to 14 weeks after delivery). Data were analyzed to compare quality of life in the two study groups.

**Results:**

In all 100 women were interviewed (50 with normal delivery and 50 with caesarean section). Postnatal quality of life in both groups was improved from time1 to time 2. However, comparing the mean scores between the normal and caesarean delivery groups the results showed that in general the normal vaginal delivery group had a better quality of life for almost all subscales in both assessment times. The differences were significant for vitality (mean score 62.9 vs. 54.4 P = 0.03) and mental health (mean score 75.1 vs. 66.7, P = 0.03) at first assessment and for physical functioning (mean score 88.4 vs. 81.5, P = 0.03) at second evaluation. However, comparing the findings within each group the analysis showed that the normal vaginal delivery group improved more on physical health related quality of life while the caesarean section group improved more on mental health related quality of life.

**Conclusion:**

Although the study did not show a clear cut benefit in favor of either methods of delivery that are normal vaginal delivery or caesarean section, the findings suggest that normal vaginal delivery might lead to a better quality of life especially resulting in a superior physical health. Indeed in the absence of medical indications normal vaginal delivery might be better to be considered as the first priority in term pregnancy.

## Background

### The problem

The extent of postnatal morbidity in vaginal delivery and caesarean section has increasingly been recognized in recent years [1]. The focus on obvious morbidity such as anemia, infections and hemorrhage has been widened to include other areas such as sexual functioning, backache, painful perineum and constipation. Screening for postnatal depression is also well established [2]. However, the debate on the best practice (vaginal delivery versus caesarean section) to minimize postnatal morbidity still is a matter of controversy both from professionals' perspectives [3] and from women's perceptions of the childbirth experience [4]. Discussion about such issues goes beyond the scope of this paper but concerns about increase in caesarean section rates remain unresolved, yet this increase is not associated with improvement in postpartum mortality or morbidity [5]. A wide variation exists among countries worldwide, ranging from 0.4 to 40 percent of all deliveries performed [6]. A study from Brazil with a high rate of caesarean section found that doctors frequently persuaded their patients to accept a scheduled caesarean section for conditions that either did not exist or did not justify this procedure. The study suggested that the problem identified in Brazil may extend well beyond Brazil and should be of concern to those with responsibility for ethical behavior in obstetric [7]

### Caesarean section in Iran

In Iran the elective caesarean section has been increasing at alarming rate and about 60% of women prefer to have caesarean to avoid labor pain or to determine the exact time of childbirth. A study from Tehran (the capital) in 1999 reported that the rate for caesarean section was between 14.6 to 39.2 in 6 teaching hospitals and 78.5 in 2 private hospitals [8]. Similarly a study comparing teaching and private maternity hospitals in year 2001 showed that the rates for elective caesarean section were 47% and 84%, respectively [9]. Of these, 14% in teaching hospitals and 86% in private hospitals were performed due to maternal request [10]. Also a recent study on caesarean section rates in teaching hospitals in Tehran found that elective caesarean section rate has been doubled during a five-year period (6.2 in 1999 to 11.8 in 2003). Overall, the caesarean section increased from 35.4% of deliveries in 1999 to 42.3% in 2003 [11].

### Comparative postnatal quality of life studies

Studies on either postnatal quality of life in general or studies that compare quality of life in new mothers after different types of delivery are limited [12]. An investigation on psychometric evaluation of health-related quality of life measures in women after different types of delivery showed that women with vaginal delivery had better health-related quality of life compared with elective or emergency caesarean section [13]. In particular comparing health-related quality of life between three modes of delivery (vaginal, elective, and emergency caesarean) it was found that patients after vaginal delivery had higher mean physical health-related quality of life scores than after caesarean section, while mean mental health-related quality of life were similar among three groups [14]. In contrast, some investigators showed that in addition to variables such as the occurrence of pregnancy complications, life stress and less social support, caesarean delivery is predictor of poorer mental health in postpartum women [15]. However, there is currently no instrument available for measuring the mothers' health-related quality of life in relation to the mode of delivery, although recently the Mother-Generated Index (MGI) was developed to identify the areas of lives that are of most concern to mothers' quality of life [16,17] or the Maternal Postpartum Quality of Life (MAPP-QOL) questionnaire that intend to measure quality of life during the early postpartum period [18,19]. The aim of this study was to examine whether postnatal health-related quality of life differed among women after different types of delivery. It was thought this might help to provide information for evidence-based practice and assist women for informed decision-making. We used a generic health-related quality of life instrument to measure quality of life after normal delivery and caesarean section in a group of Iranian women.

## Methods

### Design

This was a prospective study of quality of life of women living in Isfahan (a famous and historical city in the central part of Iran), and admitted for delivery in Isfahan health centers, affiliated to Isfahan University of Medical Sciences. In all a consecutive sample of 130 women were approached during their antenatal care and agreed to take part in the study after childbirth. Applying inclusion and exclusion criteria 100 women (50 with normal delivery and 50 with caesarean section) entered into the study and no one were excluded after entering the study. The recruitment was not based on the power calculation, and it was done post-hoc. Inclusion criteria were: being aged between 20 to 40 by the time of delivery, having one or two children, experience of just one type of delivery method, having a maximum of one abortion in the medical history and receiving prenatal care. Exclusion criteria were: having history of dystocia or instrumental delivery, still birth, having diseased or handicapped child, giving birth to a child with a weight of less than 2500 grams, history of general medical conditions, disabilities, depression, drug intake, major psychological problems, having stress -inducing experiences such as lose of a family member, divorce, or family problems. Also, those with medical conditions such as low back pain, chronic constipation, urination problems, and breast problems before pregnancy were excluded from the study. A trained female nurse at two points in time collected quality of life data: time 1 (six to eight weeks after delivery), time 2 (12 to 14 weeks postpartum). Normal delivery was defined as non-instrumental vaginal delivery and the type of caesarean section included both emergency and elective caesareans.

### Measures

Since at the time of this study the Iranian versions of postnatal quality of life measures such as the MGI or the MAPP-QOL were not available, quality of life was measured using the Iranian version of Short Form Health Survey (SF-36). It is a well-known generic health related quality of life instrument and translated into a variety of languages. It measures eight health related concepts: physical functioning, role limitation due to physical problems, bodily pain, general health perceptions, vitality, social functioning, role limitation due to emotional problems, and perceived mental health. The scores on each subscale range from 0 to 100 with higher scores indicating a better condition. The validity of the Iranian version of the SF-36 is well documented [20]. In addition, demographic data were collected using a short questionnaire during antenatal period and included recording of age, educational level, employment status, and number of children as a proxy of childbirth experiences.

### Statistical analysis

Descriptive statistics including numbers, proportions, mean and standard deviations were used to present data. Quality of life was compared between women after normal delivery and caesarean section. T-test was used for group comparisons.

### Ethics

The study received ethical approval from the Khorasgan Azad University and the Isfahan Health Authorities. All participants gave their oral consent.

## Results

In all 100 women were interviewed. The characteristics of the women in the two groups are shown in Table 1. There were very similar in their characteristics.

**Table 1:** The characteristics of women in two groups

	**Normal delivery (n = 50)**	**Caesarean section (n = 50)**
	**No. (%)**	**No. (%)**

**Age groups**		

20–24	32 (64)	31 (61)

25–29	16 (32)	17 (34)

30–34	1 (2)	2 (4)

35 ≥	1 (2)	0 (0)

Mean (SD)	24.8 (3.68)	24.7 (3.17)

Educational status*		

Illiterate	2 (4)	0 (0)

Primary	11 (22)	13 (26)

Secondary	30 (60)	28 (56)

Higher	7 (14)	9 (18)

**Employment**		

Housewife	44 (88)	42 (84)

Employed	6 (12)	8 (16)

**Number of children**		

One	26 (52)	31 (62)

Two	24 (48)	19 (38)

The women's scores on the SF-36 at 6 to 8 weeks after delivery are shown in Table 2. The analysis indicated that in all subscales the normal delivery group showed a better condition except for the general health subscale. These differences were statistically significant for the vitality (P = 0.03) and the mental health (P = 0.03).

**Table 2:** Comparing quality of life in women with normal delivery and caesarean section at 6 to 8 weeks postpartum

	**Normal delivery (n = 50)**	**Caesarean section (n = 50)**		
	**Mean (SD)**	**Mean (SD)**	**95% CI for the difference**	**P**

**Physical functioning**	79.5 (23.0)	77.2 (18.7)	10.6 to -6.0	0.58

**Role physical**	42.5 (37.2)	33.0 (33.6)	23.5 to -4.5	0.18

**Bodily pain**	62.8 (20.1)	62.1 (20.4)	8.7 to -7.2	0.85

**General health**	73.1 (19.0)	77.8 (15.0)	2.1 to -11.4	0.17

**Vitality**	62.9 (17.6)	54.4 (22.0)	0.56 to 16.4	0.03

**Mental Health**	75.1 (16.8)	66.7 (21.7)	0.67 to 16.1	0.03

**Role emotional**	50.6 (45.3)	38.0 (37.5)	29.1 to -3.8	0.13

**Social functioning**	68.2 (19.6)	63.2 (23.2)	13.5 to -3.5	0.24

Comparing scores at 12 to 14 weeks postpartum the results showed that the caesarean group had slightly higher scores in vitality and social functioning whereas for other subscales the normal delivery group scored higher. The difference was statistically significant for physical functioning, P = 0.03 (Table 3).

**Table 3:** Comparing quality of life in women with normal delivery and caesarean section at 12 to 14 weeks postpartum

	**Normal delivery (n = 50)**	**Caesarean section (n = 50)**		
	**Mean (SD)**	**Mean (SD)**	**95% CI for the difference**	**P**

**Physical functioning**	88.4 (14.0)	81.5 (18.5)	0.36 to 13.4	0.03

**Role physical**	59.5 (39.4)	50.0 (38.4)	24.9 to -5.9	0.22

**Bodily pain**	71.9 (17.2)	70.7 (19.0)	8.3 to -6.0	0.75

**General health**	75.7 (18.4)	73.8 (18.2)	9.2 to -5.3	0.60

**Vitality**	61.1 (20.1)	64.3 (22.1)	5.1 to -11.5	0.45

**Mental Health**	74.7 (17.7)	72.3 (20.1)	9.9 to -5.1	0.52

**Role emotional**	62.0 (41.5)	60.6 (40.7)	17.6 to -15.0	0.87

**Social functioning**	70.5 (19.3)	71.5 (22.0)	7.2 to -9.2	0.81

To compare the findings within each group the analysis showed that the normal vaginal delivery group showed more improvements on physical health related quality of life while the caesarean section group showed more improvements on mental health related quality of life. These were just significant for the general health subscale in favor of the normal delivery group (P = 0.05) and highly significant for the vitality subscale in favor of the caesarean section group. The results are shown in Table 4.

**Table 4:** The mean score differences within each group (time 2 scores minus time 1 scores)*

	**Normal delivery (n = 50)**	**Caesarean section (n = 50)**	
	**Mean Difference (SD)**	**Mean Difference (SD)**	**P**

**Physical functioning**	8.9 (24.2)	4.3 (24.2)	0.34

**Role physical**	17.0 (40.8)	17.0 (40-8)	1.0

**Bodily pain**	9.0 (22.9)	8.6 (21.5)	0.92

**General health**	2.5 (17.4)	-4.0 (16.0)	0.05

**Vitality**	-1.8 (22.1)	9.9 (16.3)	0.003

**Mental Health**	-0.4 (15.1)	5.6 (16.5)	0.06

**Role emotional**	11.3 (46.9)	22.6 (50.5)	0.24

**Social functioning**	2.3 (22.9)	8.3 (25.0)	0.21

* Positive values indicate improvements and negative values indicate deteriorations. Higher positive values indicate more improvements and higher negative values indicate more deterioration.

## Discussion

There are many studies that assess different problems resulting from normal vaginal delivery and caesarean section, but a few studies have focused on women's health-related quality of life *pre se*. Thus, the findings of this study, although limited, could contribute to the existing literature and a better understanding of maternal health care outcomes.

We showed that there were differences between health-related quality of life among women after normal vaginal delivery and caesarean section. At first assessment (6–8 weeks postpartum) women after vaginal delivery significantly scored higher on the mental health and the vitality subscales compared to new mothers after caesarean section. Although these differences disappeared in the second assessment (12–14 weeks postpartum), the findings indicate that in the short-term vaginal delivery might be preventive of postnatal depression. There is a wide range of prevalence of postnatal depression among women from different countries. A recent review of 143 studies from 40 countries demonstrated that reported prevalence of postnatal depression ranged from almost 0% to 60% [21]. Postnatal depression is associated with problems in the mother-infant relationship, which in turn have an adverse effect on the course of child cognitive and emotional development [22]. However, recent evidence does not support significant differences in postpartum depression between women who had normal vaginal delivery or caesarean section [23,24]. In addition, as suggested postpartum quality of life may be influenced by factors other than type of delivery, such as mother-related factors (for example amount of blood loss, duration of gestation, first delivery or not, presence of co-morbid conditions) and child-related factors (for example the condition of the baby such as his or her health condition, gender, and weight) [13].

It is argued that postpartum mothers experience certain physical health problems that may affect their quality of life, future health, and health of their children. Yet, the physical health of postpartum mothers is relatively neglected in both research and practice [25]. In our study at second assessment (12–14 weeks postpartum) women after normal vaginal delivery showed significant higher physical functioning. This is consistent with recent findings by other investigators where in a study of 141 new mothers it has been shown that the average period to reach full physical recovery was 3 weeks for vaginal delivery, 6 weeks for elective caesarean section, and more than 6 weeks for emergency caesarean section [14].

In three areas women after caesarean section scored higher (better) on the SF-36. These were general health at first assessment and vitality and social functioning at second evaluation (Table 2 and Table 3). We do not know why this occurred but possibly one might argue new mothers after caesarean section received more hospital care and thus showed a slightly better condition on these two subscales. A review of the literature indicated that a small numbers of women would request a caesarean section and this request is influenced by a range of personal or societal reasons including perceived inequality and inadequacy of care after vaginal delivery [5]. Perhaps this means that new mothers after caesarean section receive more adequate care compared with care after vaginal delivery. However, since in our study mothers in the caesarean section group consisted of both emergency and elective caesarean section, therefore one might argue the findings were influenced by the fact that women with elective or emergency caesarean may experience rather different quality of life during postnatal period [13,14,26,27]. Even this might explain the observed within group differences (Table 4).

Caesarean section is not simply a mode of childbirth, it is also an operation, and like any form of surgery, particularly emergency surgery, can cause health problems. Caesarean delivery increases the incidence of surgical intervention and problems resulting from hospitalization. It also makes financial pressure on family and society. A study comparing early postpartum sleep and fatigue for mothers after caesarean section and vaginal delivery found that mothers with vaginal delivery had less hospitalization and more total sleep time [28]. In this study we also found that overall mothers in normal delivery group reported a better health related quality of life and slightly scored higher (better) on the SF-36 questionnaire.

This was a small size study and thus the results should not be generalized. It is unlikely to reach to a general conclusion from such a small study. It seems that still there is a need to carry out more robust and larger studies to find out which types of delivery exactly could improve quality of life in new mothers. The future studies also should consider variables that are related to social and cultural research environment where the potential studies would be carried out. In addition we recommend the future studies include both general and specific measures in assessing postnatal quality of life among women. Unfortunately we only used a general instrument and this might be considered as a limitation.

## Conclusion

Although the study did not show a clear cut benefit in favor of either methods of delivery that are normal vaginal delivery or caesarean section, the findings suggest that normal vaginal delivery might lead to a better quality of life especially resulting in a superior physical health. Indeed in the absence of medical indications normal vaginal delivery might be better to be considered as the first priority in term pregnancy.

## Competing interests

The authors declare that they have no competing interests.

## Authors' contributions

BT was the main investigator, and collected the data and wrote the first draft. SP and ML contributed to the study design and interpretation of the findings. AK contributed to the statistical analysis. AM analyzed the data, and wrote the final version of the paper. All authors read and approved the final manuscript.

## Pre-publication history

The pre-publication history for this paper can be accessed here:


